# Effect of dexmedetomidine-etomidate-fentanyl combined anesthesia on somatosensory- and motor-evoked potentials in patients undergoing spinal surgery

**DOI:** 10.3892/etm.2014.1555

**Published:** 2014-02-18

**Authors:** SHENG LIN, NA DAI, ZHENGYAN CHENG, WEI SHAO, ZHIJIAN FU

**Affiliations:** 1 Department of Anesthesiology, Yantai Shan Hospital, Yantai, Shandong 264001, P.R. China; 2 Shandong University, Jinan, Shandong 250001, P.R. China; 3 Public Management Department, Yantai Vocational College, Yantai, Shandong 264001, P.R. China; 4 Department of Anesthesiology, Shandong Provincial Hospital of Shandong University, Jinan, Shandong 250001, P.R. China

**Keywords:** anesthesia, general anesthetics, intravenous, evoked potentials, medetomidine

## Abstract

This aim of the present study was to evaluate the effects of dexmedetomidine (DEX) on the intraoperative monitoring of somatosensory-evoked potentials (SEPs) and motor-evoked potentials (MEPs) in patients undergoing spinal surgery. A total of 36 patients who received spinal surgery under general anesthesia were randomly divided into two groups (n=18 per group), group C, the test group and group D, the control group, and these groups were subjected to a matching anesthesia induction. In brief, the anesthesia was administered via injection of etomidate and fentanyl; once the patients were unconscious, a laryngeal mask airway (LMA) was inserted, SEPs and MEPs were monitored and the collected data were considered to be basic data. Cisatracurium was subsequently injected and an endotracheal tube (7#) was inserted to replace the LMA. The following procedures were conducted for anesthesia maintenance: Group C, the anesthesia was maintained via target-controlled infusion of etomidate and intermittent injection of fentanyl; and group D, DEX (0.5 μg/kg) was injected over a duration of 10 min and then pumped at a rate of 0.5 μg/kg/h. In the two groups, all of the other drugs used were the same and a muscle relaxant was not administered. The bispectral index was maintained between 45 and 55 during surgery, and the SEPs and MEPs were monitored continuously until the surgery was completed. No significant difference in duration and amplitude of the SEPs (P15-N20) was identified between group C and D (P>0.05). Furthermore, the MEPs were monitored in the two groups at specific durations and no significant difference was observed between the two groups (P>0.05). The SEPs and MEPs were maintained in the patients who were administered with the DEX-etomidate-fentanyl combined anesthesia during spinal surgery.

## Introduction

The spinal cord or a spinal nerve can be injured during spinal surgery. To avoid this adverse effect, surgeons monitor intraoperative somatosensory-evoked potentials (SEPs) and motor-evoked potentials (MEPs) (1,2). Total intravenous anesthesia is commonly administered in patients for surgery. For example, dexmedetomidine (DEX), a specific agonist of the α2-adrenergic receptor, elicits a strong sedative effect without any evident respiratory depression and is administered as an anesthetic adjuvant. However, no studies have been conducted that determine the effect of DEX-etomidate-fentanyl combined anesthesia on SEPs and MEPs. In the present study, it was hypothesized that the combined anesthesia would not affect SEPs or MEPs in patients that were undergoing spinal surgery.

In the present prospective study, we observed the change of intraoperative SEPs and MEPs when the patients were anesthetized by dexmedetomidine-etomidate-fentanyl. We hope this result could be used to find clues to a proper anesthesia in ACDF surgery.

## Patients and methods

### Patients and groups

A total of 36 patients [American Society of Anesthesiologists physical status classification system category I–II (3), aged 18–60 years, muscle strength III–V] who underwent anterior cervical discectomy and fusion by general anesthesia, were randomly divided into two groups (n=18 per group) according to a random number table: Group C, control group (etomidate + fentanyl anesthesia) and group D, test group (DEX + etomidate + fentanyl anesthesia group). This study was conducted in accordance with the declaration of Helsinki and approval was obtained from the Ethics Committee of Shandong University (Jinan, China). Written informed consent was obtained from all participants. In all of the cases, gender and height were not considered and the following patients were excluded: Patients who suffered from a nerve conduction pathway injury, intracranial hypertension syndrome, diabetic peripheral neuropathy or myasthenia gravis. Furthermore, all of the patients were blinded to the interventions.

### Anesthesia methods

Phenobarbital sodium (0.1 g) and atropine (0.5 mg) were injected intramuscularly 30 min prior to the induction of anesthesia. The electrocardiogram and oxygen saturation (PETCO_2_) of the patients were routinely monitored and the invasive arterial pressure was monitored via the radial artery. Approximately 0.9% NaCl and 6% hydroxyethyl starch were injected into the peripheral vein of the patient at a ratio of 1:1 and the infusion rate was adjusted according to their hemodynamics until a constant rate was reached; the constant rate was maintained until completion of the surgery. Similar anesthesia induction methods were used in the two groups, according to the following procedures: Etomidate was applied using a target-controlled infusion (TCI) of Arden-model (Si Lugao High-tech Development Co., Ltd., Beijing, China) at an effect-site concentration in the plasma of 0.5–1.0 μg/ml. In addition, ~1.5–2 μg/kg fentanyl was administered and once the patients were unconscious, a laryngeal mask airway (LMA; Zhuhai Funiya Medical Equipment Co., Ltd., Zhuhai, Guangdong, China) was inserted to maintain ventilation. The SEPs and MEPs were monitored using an Endeavor CR 16 neurophysiological detector (Thermo Nicolet Corporation, Beijing, China) and the collected data were considered to be the basic data. Subsequently, cisatracurium was injected and an endotracheal tube (7#) was inserted to replace the LMA.

### Anesthesia maintenance

In group C, the level of anesthesia was maintained using a TCI of etomidate of 0.3–0.8 μg/ml (lot no. 32022379; Jiangsu Hengrui Medical Co., Ltd., Lianyugang, China) and fentanyl was intermittently injected into the patients. In group D, 0.5 μg/kg DEX (lot no. 10082534; Jiangsu Hengrui Medical Co., Ltd.) was injected into the patients for 10 min and pumped at 0.5 μg/kg/h into a TCI-III pump (Beijing Si Lugao High-tech Development Co., Ltd.). Additional drugs that were administered in group D were the same as in group C and muscle relaxant was not administered in either of the groups. All of the patients were subjected to controlled ventilation at a frequency of 12 bpm, the oxygen frequency ranged between 1 and 5 ml/min and the ratio of inspiration to expiration was 1:2. During surgery, the patient’s PETCO_2_ of the patients was maintained between 35 and 45 mmHg and the bispectral index (BIS) was maintained between 45 and 55.

### SEP monitoring

The SEPs were monitored using an Endeavor CR 16 neurophysiological detector. The initial SEP signals (pre-baseline) were obtained after the LMA was inserted and the baseline signals were obtained prior to surgery. Continuous upper and lower extremity stimulation was performed simultaneously throughout surgery at intervals of 10 min until the surgery was completed. Stimulation was accomplished using square-wave electrical pulses under the following conditions: Duration, 0.3 msec; intensity, 10–15 mA and frequency, 5.1 Hz. Somatosensory-evoked responses were monitored following bilateral median and ulnar nerve stimulation induction at the wrist. In addition, posterior tibial nerve stimulation was induced at the ankle using subdermal needle electrodes (lot number:160331323, Carefusion Ltd., San-Diego, WI, USA). The evoked potentials were recorded referentially and differentially from multiple scalp electrodes (using the international 10–20 montage system; Cz, C3, C4 and Fpz) in addition to a linked ear electrode. The filter bandwidth was 10–500 Hz and scanning was conducted at 100 msec superimposing 100 times; the SEP amplitudes were defined as the peak-to-peak amplitude.

### MEP monitoring

Multi-pulse transcranial electrical stimulation was generated using the Endeavor CR 16 neuro-physiological detector during the MEP monitoring. The pre-baseline MEP signals were obtained following induction of the LMA and the baseline MEP signals were obtained when T4/T1 (Train of Four stimulations test, muscle relaxation monitoring) were >75% using the Endeavor CR 16. Stimulation was applied using short trains of four square-wave, monophasic, anodal and constant-current electrical pulses at a duration of 500–1,000 msec with an inter-stimulus interval of 2 msec in sites located at 2 cm anterior to the C1/C2 position of the international 10–20 system. The stimulus intensity ranged between 200 and 400 V. The MEPs were recorded simultaneously from the abductor hallucis, anterior tibialis and abductor pollicis brevis muscles bilaterally using subdermal needles with a distant reference electrode.

### Monitoring criteria of SEPs and MEPs

The SEP waveforms were analyzed to determine the latency and peak-to-peak amplitude. Critical SEP changes were defined as a decrease in amplitude of >50% or an increase in latency of >10% (from the baseline values) and a positive result was defined as a bilateral or asymmetrical MEP loss. The same systemic parameters or additional potential factors, such as blood pressure and body temperature, were considered for the two groups and surgery was temporarily terminated when a positive SEP or MEP event occurred. For patients that exhibited a positive result, surgery was reviewed to determine whether or not an intraoperative intervention had occurred; moreover, DEX administration was terminated. When a waveform could not be continually recovered, a wake-up test was conducted and the case was excluded from the study.

### Data recording

Heart rate (HR) and mean arterial pressure (MAP) were recorded at the following time points: 1 min prior to induction of anesthesia (pre-baseline, T0); 1 min following LMA insertion (baseline, T1); 10 min (T2); 30 min (T3) and 60 min (T4) following DEX administration. The amplitudes and latency of the SEPs (P15-N20) were recorded at T1–4. In addition, the positive event number of MEPs was recorded as follows: Period 1, from the time point following LMA induction to the time point prior to conduction of endotracheal intubation; period 2, from the point at which muscle relaxation was monitored (T4/T1 >75%) to the time of surgery completion.

### Statistical analysis

The data were analyzed using SPSS 11.0 (SPSS Inc., Chicago, IL, USA) and expressed as means ± standard deviation. The group t-test was used to compare the differences between groups and repeated measures analysis of variance was used to compare intra-group differences. The χ^2^-test was used to compare the differences between enumerated data and P<0.05 was considered to indicate a statistically significant difference.

## Results

### General data

A total of 36 patients were randomly divided into two groups that received specific treatments. However, one patient in group D was excluded from the study due to the loss of SEP and MEP waves resulting from the surgery. Therefore, only 17 participants were included in group D. In the two groups, no significant difference was observed in gender, age, height or operation time (P>0.05; Table I).

### Comparison of etomidate, fentanyl dosage and hemodynamics

The etomidate and fentanyl dosages in group D decreased compared with group C (P<0.05, Table I). Following administration of DEX, patient HR decreased at each time point in group D compared with that of the group C patients (P<0.05). In addition, no significant difference was observed in MAP between the two groups (P>0.05; Table II).

### Comparison of SEPs and MEPs

No significant difference was observed in the amplitude and latency of the SEP waves (P15-N20) in group D compared with group C (P>0.05; Table III).

The positive event number of MEPs was monitored at the specified time points in the two groups, however, no significant differences were observed (P>0.05; Table IV).

## Discussion

Intraoperative SEP and MEP monitoring is recommended during spinal surgery as the spinal cord or the nerve roots are at risk of injury. Combined SEP and MEP monitoring provides an improved monitoring sensitivity during spinal surgery and is capable of predicting neurological injury (4,5). Therefore, combined SEP and MEP monitoring was performed in the present study. The SEPs and MEPs were monitored prior to performance of endotracheal intubation to remove the patient variables while the baseline data were obtained.

Previous studies identified that etomidate anesthesia may provide stable intraoperative neurophysiological monitoring (6–8). In addition, as an anesthetic adjuvant, DEX attenuates the changes in hemodynamics during anesthesia induction and decreases myoclonus, which results from the administration of etomidate (9).

In the present study, the depth of anesthesia was maintained at the same level of BIS (45–55). A previous study identified that DEX exhibits greater selectivity to the α2-adrenergic receptor compared with clonidine (10) and elicits an effect that is eight times greater than that of clonidine; therefore, DEX elicits analgesic and sedative effects (11–13). In the present study in accordance with the recommended drug dosage, DEX was injected using a pump at a rate of 0.5 μg/kg/h until surgery had been completed.

A previous study observed that intravenously injected DEX reduced BIS and decreased anesthetic consumption (14). The results of the present study indicated that the use of DEX resulted in a reduced dosage of etomidate and fentanyl. Following analysis, DEX activated the α2-adrenergic receptor in the nucleus ceruleus, thus inducing stronger sedative and analgesic effects.

The present results further indicated that the combined anesthesia of DEX-etomidate-fentanyl was able to reduce HR. This result was consistent with that of previous studies (15–17) and may be due to inhibition of the central sympathetic nerve by DEX, which decelerates the sympathetic nerve tension; this indicated that the activity of the pneumogastric nerve was strong. Furthermore, DEX may activate the presynaptic α2-adrenergic receptor in the sympathetic nerve ending and inhibit the release of norepinephrine and decrease the concentration of catecholamines in the blood plasma.

The combined DEX-etomidate-fentanyl anesthesia did not affect the SEPs of the patients that were undergoing spinal surgery. SEPs are the changes in the potentials that are observed in the cerebral cortex as a result of peripheral nerve stimulation; in addition, SEPs indicate the integrity of the somatosensory conduction pathway. However, the effective target site of DEX is not in the cerebral cortex but in the nucleus locus ceruleus adjacent to the fourth ventricle (18); so it had no direct effect on SEPs that come from the cerebral cortex. The combined anesthesia did not influence the intraoperative MEPs, which may have been because DEX did not affect the muscle strength of the patients. Anschel *et al* (19) demonstrated that it was possible to maintain MEPs during surgery using a complete intravenous anesthetic regimen that included DEX and propofol (20,21). By contrast, Mahmoud *et al* (22) identified that under the stimulation conditions used for their study, DEX, as an anesthetic adjunct to propofol-based total intravenous anesthesia (TIVA) at clinically relevant target plasma concentrations (0.6–0.8 ng/ml), significantly attenuated the amplitude of transcranial electric MEPs. However, the difference was evidently influenced by the varying monitoring criteria that were used.

In conclusion, DEX-etomidate-fentanyl combined anesthesia did not appear to influence SEPs and MEPs during spinal surgery. Therefore, TIVA using combined DEX-etomidate-fentanyl may be administered safely in spinal surgery as well as in SEP and MEP monitoring.

## Figures and Tables

**Table I:** Comparison of patient characteristics and the dosage of etomidate and fentanyl, in the two groups (means ± standard deviation).

Group	Patients	Gender (male/female)	Age (years)	Height (cm)	Weight (kg)	Dose of etomidate (mg)	Dose of fentanyl (mg)
C	18	8/10	56±13	167±14	61±9	120±30	0.85±0.10
D	17	8/9	58±12	165±14	59±11	90±20^a^	0.50±0.05^a^

a P<0.05 vs. group C.

**Table II:** Comparison of HR and MAP (means ± standard deviation).

Hemodynamic marker	Patients	T0	T1	T2	T3	T4
HR (bpm)						
Group C	18	83±9	07±11b	77±12^b^	76±10^b^	77±11^b^
Group D	17	85±8	75±10^a^^b^	62±9^a^^b^	62±10^a^^b^	60±9^a^^b^
MAP (mmHg)						
Group C	18	100±12	74±15^b^	87±14^b^	81±13^b^	90±11^b^
Group D	17	101±8^b^	77±13^b^	82±12^b^	86±15^b^	92±10^b^

a P<0.05 group D vs. group C and

b P<0.05 compared with T0.

HR, heart rate; MAP, mean arterial pressure; T0, 1 min prior to induction of anesthesia (pre-baseline); T1, 1 min following laryngeal mask airway insertion (baseline); T2–4, 10, 30 and 60 min following dexmedetomidine administration, respectively.

**Table III:** Comparison of the amplitude and latency of SEP waves (P15-N20; means ± standard deviation).

Waveform characteristics	Patients	T1	T2	T3	T4
Peak-to-peak amplitude (μV)					
Group C	18	0.81±0.10	0.79±0.09	0.78±0.08	0.84±0.09
Group D	17	0.79±0.07	0.76±0.05	0.78±0.08	0.83±0.12
Latency (ms)					
Group C	18	21±3	20±4	21±2	24±3
Group D	17	22±4	22±3	20±2	20±4

P>0.05, group D vs. group C. SEP, sensory evoked potential; T1, 1 min following LMA insertion (baseline); T2–4, 10, 30 and 60 min following dexmedetomidine administration, respectively.

**Table IV:** Comparison of the positive event number of motor-evoked potentials in the two groups.

Group	N	Period 1	Period 2
C	18	0/18	0/18
D	17	0/17	0/17

P>0.05, group D vs. group C. Period 1, time point following laryngeal mask airway induction/the time point prior to conduction of endotracheal intubation; period 2, point at which muscle relaxation was monitored (T4/T1 >75%)/the time of surgery completion; T1, 1 min following laryngeal mask airway insertion (baseline); T4, 60 min following dexmedetomidine administration.

## References

[1] Schwartz DM, Auerbach JD, Dormans JP (2007). Neurophysiological detection of impending spinal cord injury during scoliosis surgery. J Bone Joint Surg Am.

[2] Hyun SJ, Rhim SC (2009). Combined motor and somatosensory evoked potential monitoring for intramedullary spinal cord tumor surgery: correlation of clinical and neurophysiological data in 17 consecutive procedures. Br J Neurosurg.

[3] Miller RD, Eriksson LI, Fleisher LA, Wiener-Kronish JP, Young WL (2011). Miller’s Anesthesia.

[4] Li F, Gorji R, Allott G, Modes K, Lunn R, Yang ZJ (2012). The usefulness of intraoperative neurophysiological monitoring in cervical spine surgery: a retrospective analysis of 200 consecutive patients. J Neurosurg Anesthesiol.

[5] Pelosi L, Lamb J, Grevitt M, Mehdian SM, Webb JK, Blumhardt LD (2002). Combined monitoring of motor and somatosensory evoked potentials in orthopaedic spinal surgery. Clin Neurophysiol.

[6] Taniguchi M, Nadstawek J, Langenbach U, Bremer F, Schramm J (1993). Effects of four intravenous anesthetic agents on motor evoked potentials elicited by magnetic transcranial stimulation. Neurosurgery.

[7] Scheufler KM, Zentner J (2002). Total intravenous anesthesia for intraoperative monitoring of the motor pathways: an integral view combining clinical and experimental data. Neurosurg.

[8] Ghaly RF, Lee JJ, Ham JH, Stone JL, George S, Raccforte P (1999). Etomidate dose-response on somatosensory and transcranial magnetic induced spinal motor evoked potentials in primates. Neurol Res.

[9] Menda F, Köner O, Sayin M, Türe H, Imer P, Aykaç B (2010). Dexmedetomidine as an adjunct to anesthetic induction to attenuate hemodynamic response to endotracheal intubation in patients undergoing fast-track CABG. Ann Card Anaesth.

[10] Virtanen R, Savola JM, Saano V, Nyman L (1988). Characterisation of the selectivity, specificity and potency of medetomidine as an α2-adrenoceptor agonist. Eur J Pharmacol.

[11] Hunter JC, Fontana DJ, Hedley LR (1997). Assessment of the role of alpha2-adrenoceptor subtypes in the antinociceptive, sedative and hypothermic action of dexmedetomine in transgenic mice. Br J Pharmacol.

[12] Jalonen J, Hynynen M, Kuitunen A, Heikkilä H, Perttilä J, Salmenperä M, Valtonen M, Aantaa R, Kallio A (1997). Dexmedetomidine as an anesthetic adjunct in coronary artery bypass grafting. Anesthesiology.

[13] Wijeysundera DN, Naik JS, Beattie WS (2003). Alpha-2 adrenergic agonists to prevent perioperative cardiovascular complications: a meta-analysis. Am J Med.

[14] Shin HW, Yoo HN, Kim DH, Lee H, Shin HJ, Lee HW (2013). Preanesthetic dexmedetomidine 1 μg/kg single infusion is a simple, easy, and economic adjuvant for general anesthesia. Korean J Anesthesiol.

[15] Martin E, Ramsay G, Mantz J, Sum-Ping ST (2003). The role of the alpha2-adrenoceptor agonist dexmedetomidine in postsurgical sedation in the intensive care unit. J Intensive Care Med.

[16] Ben-Abraham R, Ogorek D, Weinbroum AA (2000). Dexmedetomidine: a promising agent for anesthesia and perioperative care. Isr Med Assoc J.

[17] Yildiz M, Tavlan A, Tuncer S, Reisli R, Yosunkaya A, Otelcioglu S (2006). Effect of dexmedetomidine on haemodynamic responses to laryngoscopy and intubation: perioperative haemodynamics and anaesthetic requirements. Drugs R D.

[18] Stone LS, Wilcox GL (2004). Alpha-2-adrenergic and opioid receptor additivity in rat locus coeruleus neurons. Neurosci Lett.

[19] Anschel DJ, Aherne A, Soto RG (2008). Successful intraoperative spinal cord monitoring during scoliosis surgery using a total intravenous anesthetic regimen including dexmedetomidine. J Clin Neurophysiol.

[20] Tobias JD, Goble TJ, Bates G, Anderson JT, Hoernschemeyer DG (2008). Effects of dexmedetomidine on intraoperative motor and somatosensory evoked potential monitoring during spinal surgery in adolescents. Paediatr Anaesth.

[21] Bala E, Sessler DI, Nair DR, McLain R, Dalton JE, Farag E (2008). Motor and somatosensory evoked potentials are well maintained in patients given dexmedetomidine during spine surgery. Anesthesiology.

[22] Mahmoud M, Sadhasivam S, Salisbury S (2010). Susceptibility of transcranial electric motor-evoked potentials to varying targeted blood levels of dexmedetomidine during spine surgery. Anesthesiology.

